# Retinal Degeneration: A Window to Understand the Origin and Progression of Parkinson’s Disease?

**DOI:** 10.3389/fnins.2021.799526

**Published:** 2022-02-04

**Authors:** Yanyan Zhang, Xiaoguang Zhang, Yunhua Yue, Tian Tian

**Affiliations:** ^1^Department of Neurology, The First Affiliated Hospital of Zhengzhou University, Zhengzhou University, Zhengzhou, China; ^2^Department of Neurology, Yangpu Hospital, School of Medicine, Tongji University, Shanghai, China

**Keywords:** Parkinson’s disease, visual deficits, morphological changes, neuropathology, retinal imaging technology

## Abstract

Parkinson’s disease (PD), the second most prevalent neurodegenerative disorder, manifests with motor and non-motor symptoms associated with two main pathological hallmarks, including the deterioration of dopaminergic cells and aggregation of alpha-synuclein. Yet, PD is a neurodegenerative process whose origin is uncertain and progression difficult to monitor and predict. Currently, a possibility is that PD may be secondary to long lasting peripheral affectations. In this regard, it has been shown that retinal degeneration is present in PD patients. Although it is unknown if retinal degeneration precedes PD motor symptoms, the possibility exists since degeneration of peripheral organs (e.g., olfaction, gut) have already been proven to antedate PD motor symptoms. In this paper, we explore this possibility by introducing the anatomical and functional relationship of retina and brain and providing an overview of the physiopathological changes of retinal structure and visual function in PD. On the basis of the current status of visual deficits in individuals with PD, we discuss the modalities and pathological mechanism of visual function or morphological changes in the retina and focus on the correlation between visual impairment and some representative structural features with clinical significance. To consider retinal degeneration as a contributor to PD origin and progress is important because PD evolution may be monitored and predicted by retinal studies through state-of-the-art techniques of the retina. It is significant to integrally understand the role of retinal morphological and functional changes in the neurodegenerative process for the diagnosis and therapeutic strategies of PD.

## Introduction

Parkinson’s disease (PD) is a chronic and multisystemic neurodegenerative disease characterized by a series of motor symptoms (bradykinesia, resting tremor, rigidity, and postural instability) and non-motor neurologic phenomena (sleep disturbances, autonomic dysfunction, gastrointestinal, urogenital problems, cognitive decline, psychiatric symptoms, sleep abnormalities, as well as visual disturbances) (Santos Garcia et al., 2019; Xu et al., 2019). Age is a main factor of PD, and the global impact of the disease is emerging, with its prevalence at around >2% of all persons above 65 years of age, and >4% of all persons over the age of 80 years (Gbd 2015 Neurological Disorders Collaborator Group, 2017; Santos Garcia et al., 2019; Xu et al., 2019). Individuals suffering from PD are increasing and are estimated to be 12 million patients by 2050 (Ggbd 2016 Parkinson’s Disease Collaborators, 2018), and early diagnosis and intervention of PD pathology plays a very important role in medical health.

Elucidating the derivation of pathological changes is critical for the early diagnosis and intervention of PD. However, the pathological origin of PD is debated. Consistent with the dopamine depletion and pathologic a-Syn in the nigrostriatal pathway described in previous studies, the two pathologic hallmarks have observed in peripheral nervous system and various end-organs that lead to numerous non-motor manifestation of PD including autonomic impairment (Postuma et al., 2015), sleep dysregulation, mood disorder (Ortuno-Lizaran et al., 2018b) dementia, and visual alterations (Ortuno-Lizaran et al., 2018a). Increasing studies have now discussed a possibility of PD pathology initially arising outside of the central nervous system (CNS). Indeed, the spreading of a-Syn to the brain via peripheral inoculation (e.g., olfaction, gut) has been amply elucidated (Kim et al., 2019). Visual symptoms, including glaucoma (Pavlenko et al., 2018), dry eyes (Friedman, 2004; Tamer et al., 2005; Reddy et al., 2013), visual hallucinations (Onofrj et al., 2006; Williams et al., 2008), and deficits in color vision appears early in the disease in PD patients (Stenc Bradvica et al., 2015). As shown in Table 1, PD patients suffer from different eye disorders [Blinking (London et al., 2013; Seiple et al., 2016), eye movement dysfunctions (Anderson and MacAskill, 2013; Hanuska et al., 2015; MacAskill and Anderson, 2016), pupillary imbalance (Jain et al., 2011), nuclear cataract (Lai et al., 2015; Klettner et al., 2016)]. Ophthalmological examinations of subjects with PD also suggest a loss of color vision problems, visual acuity impairment as well as the deficiency of spatial contrast sensitivity (Uc et al., 2005; Bertrand et al., 2012; Weil et al., 2016; Guo et al., 2018). The cellular and molecular studies have demonstrated loss of dopaminergic amacrine cells and retinal ganglion cells are partially responsible for the reduced contrast sensitivity, impairment in visual acuity, or electroretinographic response in individuals with PD (Yenice et al., 2008; Esteve-Rudd et al., 2011; Koens et al., 2018; Ortuno-Lizaran et al., 2020). On post-mortem observation of PD patients, authors have found the loss of dopaminergic retinal cell and the aggregation of a-Syn (Diederich et al., 2014; Ortuno-Lizaran et al., 2018a). Interestingly, it has recently been proposed that animal models with retinal damage due to intravitreal injection of minimal doses of neurotoxins display symptoms of experimental PD (Willis et al., 2014). A further example is that the retinal exposure of welding flash is associated with increased incidence of PD (Willis, 2005). Conversely, an immune tolerance induced by eyes via administration of antigens into the anterior chamber could be used as a therapeutic approach to promote neuroprotection for neurodegenerative diseases (Farooq and Ashour, 2013; Toscano-Tejeida et al., 2016; Pineda-Rodriguez et al., 2017). Thus, it is reasonable to conclude that the retina may be intimately involved with the onset and progression of PD as a potential precipitating factor outside of the CNS.

**TABLE 1:** Visual dysfunctions and manifestation in PD patients.

Organ	Mechanisms	Main manifestations	Morbidity	References
①Eyelid	Frontal DAN dysfunction	(1) Blinking: Bradykinesia of voluntary blinking, Abnormalities of reflex blinking, Reduced amplitude and blink rate		Postuma et al., 2015; Gbd 2015 Neurological Disorders Collaborator Group, 2017; Ggbd 2016 Parkinson’s Disease Collaborators, 2018; Santos Garcia et al., 2019; Xu et al., 2019
		(2) Apraxia of eyelid opening		Ortuno-Lizaran et al., 2018b
		(3) Uncomfortable sensations, red eyes	53–60%	Ortuno-Lizaran et al., 2018a
		(4) Muscle disorder: eyelid retraction, eyelid ptosis, lepharospasm		Reddy et al., 2013; Kim et al., 2019
②Eyebulb	Extrapyramidal damage	(1) Eye movement dysfunctions: convergence insufficiency Abnormal saccades Smooth pursuit impairment		Friedman, 2004; Tamer et al., 2005; Onofrj et al., 2006
		(2) Diplopia	10–30%	Williams et al., 2008
③Pupil	Autonomic disorders	Pupillary imbalance: Reduced amplitude of contraction, Prolonged contraction time		Guo et al., 2018
④Lens	Mitochondrial dysfunction	Nuclear cataract	16–24%	Uc et al., 2005; Weil et al., 2016
⑤Retina	Retinopathy	(1) Visual acuity	70%	Bertrand et al., 2012; Ortuno-Lizaran et al., 2020
	DAN dysfunction	(2) Spatial contrast sensitivity		Esteve-Rudd et al., 2011
	a-Syn deposition	(3) Color vision		Esteve-Rudd et al., 2011; Willis et al., 2014
⑥Optic nerve	Macular thickness	Visual field defects	60–70%	Willis, 2005
⑦Visual cortex	Cortex impairment	(1) Visuospatial deficits	30–60%	Toscano-Tejeida et al., 2016; Pineda-Rodriguez et al., 2017
		(2) Visual hallucination		
		(3) Facial expression recognition		
⑧Other auxiliary apparatus	Retina DAN dysfunction	(1) Glaucoma	30–40%	Uc et al., 2005; Farooq and Ashour, 2013
		(2) Dry eyes	50%	Cameron et al., 2017; Satue et al., 2017; Ortuno-Lizaran et al., 2018b
		(3) Rapid eye movement sleep behavior		Garcia-Martin et al., 2014b

The retina presents a unique opportunity to study the CNS. First, it shares a common origin, structure, and physiology with the brain in terms of nervous and microvascular systems (Cameron et al., 2017). Over the past decades, investigators have attempted to access the tools that leverage the accessibility of the retina to better understand and diagnose PD. The retinal degeneration, retinal ganglion cells (RGCs) loss, and retinal thinning as well as visual disorders were observed in PD and animal models. As the only portion of CNS, furthermore, the retina is capable of reliable and precise measures of high-resolution imaging, retinal neurons, and vascular morphology therefore begin to be analyzed by ocular measurements from large studies utilizing tools. For instance, using the optical coherence tomography (OCT) imaging, some authors have found retinal nerve fiber layer (RNFL) thinning (Garcia-Martin et al., 2014b; Mailankody et al., 2015; Satue et al., 2017), and lower capillary perfusion density (CPD) and capillary afflux index in the retinal vascular morphology revealed by OCT angiography (OCTA) and fundus imaging (Guan et al., 2013; Robbins et al., 2021).

In this review, we systematically assess evidence in the field of PD with a focus on the morphological changes and visual dysfunction in the retina. We first review the anatomical and functional relationship of the retina and the brain. Also, the introduction and development of new and highly sensitive ocular technology were described. Special care is taken to discuss up-to evidences on retinal morphological alterations and visual disorders in PD patients, with the highlight of the correlation between several representative structural features and visual impairment in PD patients. Finally, we emphasized the role of some retinal morphological changes in diagnostic and prognostic progression for visual neuropathology of the neurodegenerative disease.

## Structure and Function of the Retina

In embryological origin, the retina is derived from the neuroectoderm. The retina shares a common origin and similar anatomy with brain tissue. Revisiting some of the basic anatomy of the retina is helpful for appreciating the impact of diseases on the retina.

**FIGURE 1:**
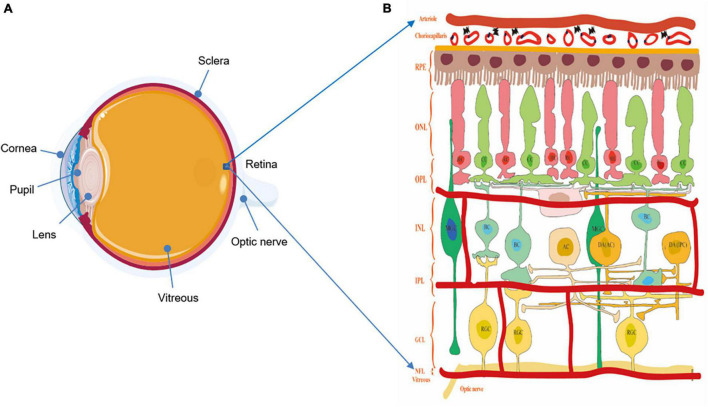
**(A)** The structure of the eyeball. **(B)** The structure of the retina and diagram of the retinal neurons. Notably, understanding the physiologic structure and function is critical for better exploring the relationship of visual function and morphological changes. The retina possesses complex and multilayer structures and a large number of cells with microcircuits features and different functions. ILM, inner limiting membrane; RNFL, retina nerve fibers layer; GCL, ganglion cell layer; IPL, inner plexiform layer; INL, inner nuclear layer; OPL, outer plexiform layer; ONL, outer nuclear layer; ORL, outer retina layer; RPE, retinal pigmented epithelium; RC, rod cell; CC, cone cell; BC, bipolar cell; HC, horizontal cell; AC, amacrine cell; DA AC dopaminergic amacrine cell; DA IPC, dopaminergic interplexiform cell; RGC, retinal ganglion cell.

Retina and optic nerve are essential parts of the neural conduction systems, which consist of different cell types and play a crucial role in visual imaging. As an innermost, light-sensitive layer of sensory tissue in most vertebrates and some mollusks, the retina possesses complex and multilayer structures and many cells with microcircuits features and different functions (Figure 1). Morphologically, five classes of neuronal cells play the important role in shaping the structure of the retina, encoding visual information and regulating vision function. These component cells include photoreceptors (rods and cones), horizontal cells (HCs), bipolar cells (BCs), amacrine cells (ACs), and RGCs. In the normal condition, the light energy is converted to membrane potential changes in rod (RC) and cone (CC) photoreceptors in the outer retina layer (ORL). Within the outer plexiform layer (OPL), the photoreceptors convey light information to BCs under the modulation of HCs. Then, as a sole output neurons of the retina, RGCs within the inner plexiform layer (IPL) contact BC and ACs at the inner nuclear layer (INL), projecting their axons to higher visual centers (Archibald et al., 2009; Schmidt et al., 2011). Aside from the above vertical and horizontal cell bodies, there are cells or related neurotransmitters mediating visual information, including the retinal pigment epithelium (RPE) with the capacity of visual pigment regeneration, and Müller glial cells (MGC) involved in neuronal metabolism, synaptic pruning, and neurotrophy (Vecino et al., 2016). These cells mediate retinal signaling in vertical and horizontal directions, and these are vital in shaping color vision, spatial resolution, and vision sensitivity (Weil et al., 2016).

### Dopaminergic Neurons in the Retina

Malmfors (1963) first described the role of catecholamines in rat retinal function and regulation, involving in the light/dark adaptation and reshaping retinal circuitries. Subsequently, dopaminergic neurons have been identified in human retina, and overlap with neighboring DA cells as well as other retinal cells (the cone-rod, horizontal cells, and ganglion cells) (Frederick et al., 1982). In vertebrate retina, DA neurons contact two other types of amacrine cell (AII and A17) and interplexiform neurons (IPC) (Figure 1). The amacrine cells, via gap junctions, receive input from BCs and pass visual information to RGCs and the same cell types, modulating visual processing of the flow of photoreceptors-driven visual information. Compared with the AII amacrine cells, in scotopic conditions, the A17 amacrine cell receives GABAergic inputs instead of excitatory glutamatergic inputs from rod bipolar cells, and it plays a role in converging rod signals and amplifying the effects of low light stimulation (Hillman et al., 1995). DA cells are stimulated to release functional dopamine as an essential neuromodulator in photopic conditions, making synaptic contacts between DA cells and affecting on gap junction permeability both at the level of photoreceptors interactions with HCs and at the level of DA cells communication (Cameron et al., 2009; Zhang et al., 2014). In return, DA cells alter the action potential firing rate of their own cells and regulate DA release when receive excitatory or inhibitory feedback information.

In addition to this excitatory and inhibitory feedback system, the modulation of retinal DA cells conditions and dopamine concentration has diurnal variation, with low levels at night and higher levels during the day. From functional perspectives, some DA cells are light-evoked, involving in regulating the light/dark adaptation and electrophysiological communication between retinal cells in different layers depending on the circadian rhythm (Cohen et al., 1992). Also, some dopaminergic cells can activate spontaneously and alter DA level in the darkness. In turn, the DA effects on photoreceptor cells, BCs, ACs, and HCs (Popova, 2014), reshaping photomechanical movements and survival, to enhance flicker response of retinal rod pathway (Hampson et al., 1992), and regulate visual stimuli of cells communication as well as protects RNFL (Yavas et al., 2007). Knowledge of these anatomical connections and visual progressing demonstrated that DA is a chemical neurotransmitter in the retina, promoting synaptic effects and visual information to regulate electrical activity and retinomotor movements.

### Ganglion Cells and Retinal Nerve Fiber Layer

In retinal physiology, RGCs, the output neurons that project visual information from the inner retina to the brain, extend to the lateral geniculate nucleus (LGN) via a nerve fiber tract complete with an oligodendrocytic myelin sheath (Henderson et al., 2008). There are numerous subtypes of retinal cells, such as photoreceptors, HCs, BCs, and ACs, making synaptic contact with RGCs in the inner plexiform layer through different communication systems, including acetylcholine, dopamine, glutamate, glycine, and gaba-aminobutyric acid. Influenced by light, RGCs receive photosensitive information through either direct or indirect circuitry, and act as the final common pathway in the flow of visual information to the optic nerve and brain cortex (Figure 1). Sparkly, the melanopsin-containing retinal ganglion cells (mRGCs), accounting for about 0.3–0.8% of the total ganglion cells within the retina, represent a specialized class of RGCs that respond to light without rod and cone information input (Hattar et al., 2002). Some authors therefore think that mRGCs constitute a third class of photoreceptors and are directly photosensitive. In addition, mRGCs are also responsible for the non-image forming pathways, mediating the circadian rhythm and pupil constriction that are involved in mood and sleep behaviors (Güler et al., 2008). Previous studies demonstrated that GCL thinning is relevant to lower visual acuity, contrast sensitivity loss, and color deficiencies.

The RNFL is the inner most layer of the retina and is composed largely of axons of RGCs. Many studies investigated the ganglion cells’ death is inevitably reflected on thinning RNFL thickness, relatively presenting the number of RGCs axons loss (Henderson et al., 2008). Recently, the OCT and OCTA have been used in the investigation of structural changes and measures of the vertical retinal layers in the retina *in vivo*. RNFL thinning demonstrated the dopaminergic neuronal loss and decreased axons of RGCs, effecting retinal neuronal processing and electrophysiological function (Bodis-Wollner et al., 2014a).

### Microvascular and Choroidal Structure in the Retina

In addition to retina nervous systems, evidence has implicated retinal small vessel plays an important role in structural and functional changes in the retina. Based on the potential risk the role of cerebral small vessel disease for the development of PD plays (Guan et al., 2013), some studies demonstrated retinal microvascular changes have been studied retinal capillary plexus vessel density (VD) and perfusion density (PFD) as well as structural changes in PD (London et al., 2013; Robbins et al., 2021). Thus, structural changes in retinal microvascular are seen as non-invasive biomarkers for the disease detection.

Considering the common embryologic and anatomic characteristics of retinal vascular with the cerebral circulation, microvascular changes in the retina may correlate with vascular changes in the CNS. The retinal vasculature is a window *in vivo* non-invasive assessment of microvasculature in the body (Figure 1). In embryology, the ophthalmic artery originates from the internal carotid artery gives off the central retinal artery, providing nutrients and oxygen to the inner layer of the retina. Metabolic waste and carbon dioxide from the retina are excreted into the sinus via the central retinal vein through the superior ocular vein. Central retinal arteries and veins form a terminal branch retinal circulation network on the surface of the retina. In addition, microvasculature of the retina shares similar neurobiology and electrophysiological function with those in CNS (Ge et al., 2021).

## Morphological and Functional Technologies in the Retina

As the eye is an extension of the brain, the retina displays similarities to the brain in anatomy, functionality, and pathological responses to environmental insult. So, to detect retinal morphological parameters of brain pathologies using imaging techniques seems reasonable.

**FIGURE 2:**
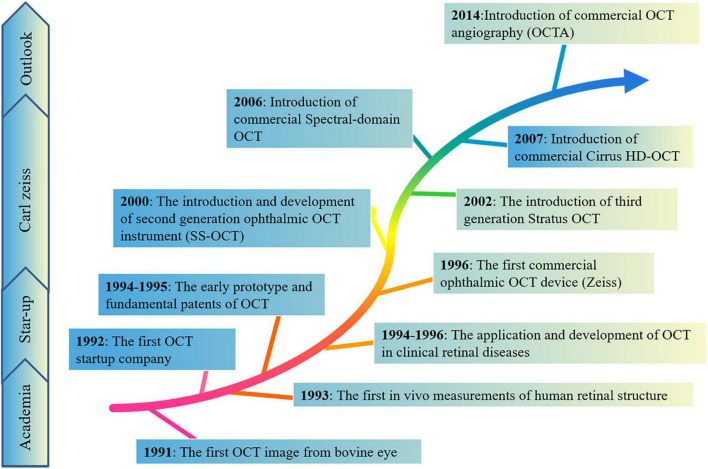
The brief historical timeline marking events elucidating morphological and technologies in the retina. These new, cost-effective, high-resolution imaging tools enabled increases in imaging speeds and quantity, further catering to the clinical needs of diagnosis and therapeutics of diseases, and increasing clinical data demonstrated the important role of OCT in diagnostic and therapeutic applications of many diseases.

Optical coherence tomography (OCT) is a non-invasive observational technique based on reflectance intensity of light, providing some real-time information of the retina on structure using infrared interferometric imaging (Figure 2). The OCT enables an optical biopsy of retina to provide two/three-dimensional cross-sectional images of the target tissue using the interference of infrared radiation (Langwinska-Wosko et al., 2016). In 1991, the first OCT image was described by David Huang in the anterior chamber of an *ex vivo* bovine eye (Huang et al., 1991). Subsequently, Fercher et al. (1993) and Swanson et al. (1993) showed the first *in vivo* measurements of human retinal structure using the non-contact and high resolution technique in 1993. Retinal OCT imaging detects and quantifies the structural correlates of these visual symptoms of patients, provides histologic level information about retinal nerve fiber layer, cells, and retinal blood vessels. With the growth of OCT scientifically and economically, OCT assesses the three-dimensional outer retina thickness in the higher image pixel density and quality of OCT (Fujimoto and Swanson, 2016). For instance, spectral domain OCT (SD-OCT) uses a wavelength of 820 nm, diminished the vitreous signal, and improved imaging of the macular choroid (Ha Usler and Lindner, 1998), reaching deeper structures of the retina. Swept-source OCT (SS-OCT) reaches deeper penetration using a wavelength of 1,020 nm. In addition, the special OCT, OCT angiography (OCTA) imaging, allows for blood flow visualization (Gulmez Sevim et al., 2019; Robbins et al., 2021). This is an emerging approach for imaging retinal vessels, can visualize microvasculature based on motion contrast from flowing blood to assess the blood pressure, intraocular pressure, vascular density of the superficial capillary plexus, deep capillary plexus, and choriocapillaris (Zhang et al., 2018). Therefore, retinal OCT imaging not only costs lower, but provides insight into the underlying pathophysiology in the earlier disease process, compared to conventional neuroimaging methods, such as the fundus color photograph, fundus fluorescein angiography, and B-ultrasonography. These new, cost-effective, high-resolution imaging tools enabled increases in imaging speeds and quantity, further catering clinical need of diagnosis and therapeutics of diseases, and increasing clinical data demonstrated the important role of OCT in diagnostic and therapeutic applications of many diseases. The OCT has become a new and highly sensitive method for detecting and analyzing some classic ocular pathologies in diseases.

Aside from the over-mentioned OsCTs, some techniques are applied to evaluating the functional performances of retinopathy of PD, including electroretinogram (ERG) and visual evoked potential (VP). The ERG reflects retinal comprehensive potential caused by a brief light stimulation recorded from the cornea. There are flash ERG and graphic ERG based on different forms of light stimulation (Netser et al., 2021). The flash ERG consists of a negative A wave, a positive B wave, and the OPs waves superimposed on the B wave. Wave A mainly reflects the hyperpolarization activity of photoreceptors, while wave B is generated by the electrical activity of MCs and BCs in the retina (Takatsuna et al., 1992; Meng et al., 2012; Normando et al., 2016). The wave OPs on the B wave are related to the electrical activity of ACs. The VEP is generated by the electrical activity in occipital cortex after the visual stimulation. The structural and functional changes in the retina cause the change of waveform amplitude and/or latency in VEP.

These techniques detect retinal nerve fiber layer (RNFL) thickness (Inzelberg et al., 2004; Hajee et al., 2009; Kirbas et al., 2013; Jimenez et al., 2014), central macular volumes, morphology in foveal vision (Pilat et al., 2016; Nunes et al., 2019), inner and outer retinal layers (Pilat et al., 2016), and retinal pigment epithelium (Uchida et al., 2018), and also assess retinal blood flow and vascular alterations as well as other pathological features of retina in PD patients. The monitoring retinal morphology and function are used for exploring hallmark signs corresponding to pathological conditions in different degrees and stages of PD.

## Pathological and Morphological Changes in Retina of Parkinson’s Disease

The retina is a simple model of the brain in the sense that some pathological impairment and morphological changes from the retina may be observed or applicable to the degenerative diseases as valuable models. In the retina of PD patients, there were dopaminergic deficiency (Schmidt et al., 2011; Vecino et al., 2016), misfolded a-synuclein (Weil et al., 2016), retinal ganglion cells loss (Malmfors, 1963), thinning of retinal nerve fiber layer (Frederick et al., 1982; Hillman et al., 1995; Cameron et al., 2009; Zhang et al., 2014), or neuroinflammatory (Cohen et al., 1992) at several levels of the visual pathway during pre-clinical stages. Moreover, studies on post-mortem of PD patients found the accumulation of misfolding α-synuclein, the main culprit of the disease, in the retinal layers, especially the OPN of patients with early PD (Hampson et al., 1992; Yavas et al., 2007; Popova, 2014). Furthermore, evidence has indicated microvasculature changes as some potential biomarkers of retinal pathological changes in subjects with PD. Compared to the control cases using immunohistochemical staining and image analysis, Guan et al. (2013) observed the decreased capillaries branching as well as shortening length and enlarging diameter in capillary network in the substantia nigra, middle frontal cortex, and other brain stem nuclei. As van der Holst et al. (2015) described, increased risk of Parkinsonism was observed in population with cerebral small vessel disease. So these structural changes of the retina of PD have been shown the association with the progression, severity, and duration of the disease (Ma et al., 2018; Hasanov et al., 2019).

### A-Synuclein Deposits

**FIGURE 3:**
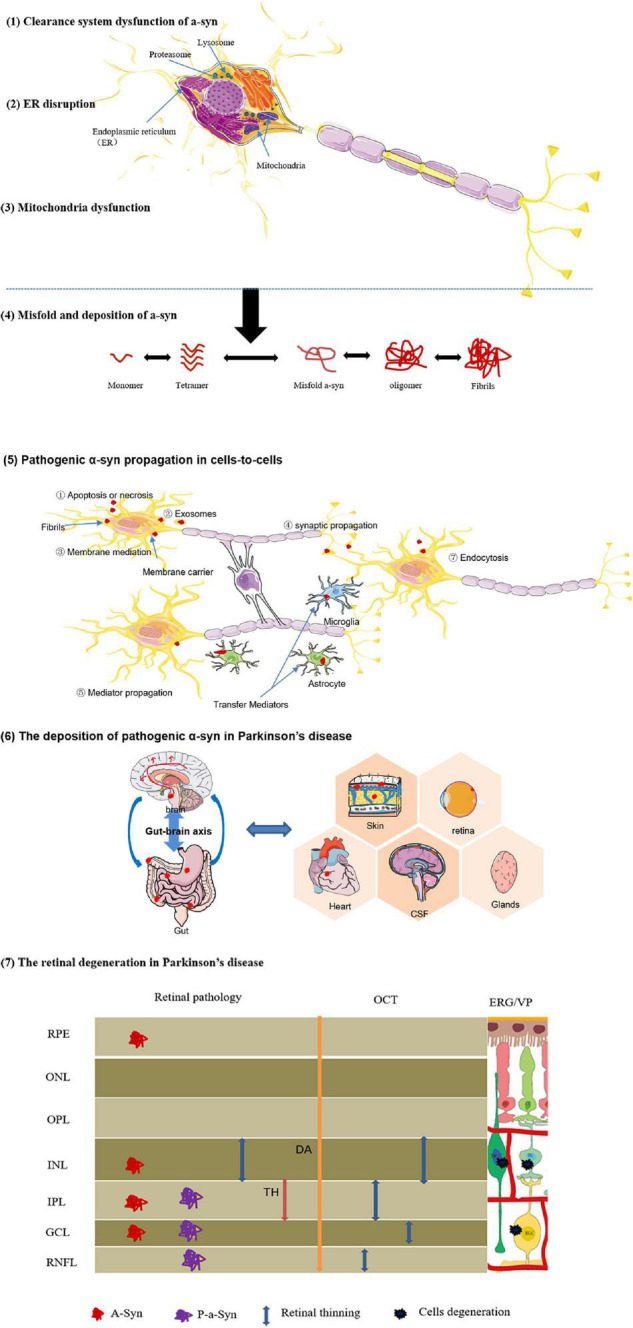
Diagrammatic interpretation of the formation, propagation, and deposition of pathogenic a-synuclein. Certain risk factors effect on neurons, and initiate some pathological mechanisms associated with the etiology of PD, including (1) protein-clearance dysfunctions, (2) ER disruption, (3) mitochondria dysfunction. These pathological effects promote (4) abnormal a-Syn misfold and deposition. (5) Intercellular prion-like transmission of pathological a-Syn. Due to the propagative mechanisms of a-Syn similar to prions, abnormal proteins are released and transmitted via different mechanisms:Apoptosis or necrosis,exosomes,membrane mediation,synaptic propagation,mediator propagation, and so on. (6) The deposition of pathogenic α-syn in Parkinson’s disease. For the definitive derivation of a-Syn, the brain-first hypothesis and gut-first hypothesis appear to be better received in current research. Especially, pathological a-Syn also was found in retina, skin tissue, heart, CSF, and glandular secretions. These pathological changes are critical for interpreting and understanding of clinical symptoms of patients with PD. (7) The retinal degeneration in Parkinson’s disease.

A-synuclein (a-Syn) is a neuropathological landmark, and its abnormal accumulation can induce neuronal death, disturbance in the dopamine mechanism, and synaptic effects (Henchcliffe and Beal, 2008). In normal physiological state, a-Syn is encoded by the SNCA gene, and belongs to the synuclein family that is involved with the exocytosis and synaptic function. In retina, a-Syn exists at the OPL, mediating membrane fusion synaptic vesicle and neurotransmitter release, fatty acid binding, cell signaling, and cell growth (Iwai et al., 1995; Burre et al., 2010; Breydo et al., 2012). In contrast, because of inducing risk factors in PD, the a-Syn protein was transformed into truncation and multimerization from monomeric and tetrameric conformation, then converted to insoluble oligomers and amyloid fibrils, and eventually perturbed dynamic equilibrium of functional a-Syn (Kahle et al., 2001; Auluck et al., 2002; Liu et al., 2005). Moreover, aberrant aggregation of a-Synuclein has prion-like properties to trigger the intercellular transmission a-Syn fibrils (PFFs); it is time-dependent and changeable, implying that the propagation of a-Syn may be the key contributor to onset and progression of PD (Willis, 2008; Willis and Freelance, 2017; Figure 3).

Similarly, the conformational or metabolic changes of a-Syn polymer, such as phosphorylated a-Syn and abnormal accumulation into insoluble aggregates are cytotoxicity for cellular and molecular metabolism (Bodis-Wollner, 2009; Stenc Bradvica et al., 2015; MacAskill and Anderson, 2016) (Table 2). Some previous studies revealed the relation between retinal a-Syn aggregates and clinical and imaging manifestations of impaired vision in PD. Bodis-Wollner et al. (2014b) reported a-synuclein aggregations in the inner retina (GCL, IPL, and INL), and observed the loss of full retinal thickness in the retina of PD. These histopathological changes in retina provide a bridge between a-synuclein inclusions and RNFL thinning detected by OCT. In addition, based on the protein inclusions in GCL associated with the impaired GCs function, authors speculated a potential route of local transmission of the anormal protein between retina and central neurons systems. Similarly, Beach et al. (2014) has found that immunopositive phosphorylated a-Syn, a specific molecular marker of synucleinopathy, presence in the inner retinal surface of PD individuals paralleling to retinal thinning (GCL, IPL, and INL) has been reported with OCT (Altintas et al., 2008; Bodis-Wollner et al., 2014b). Further, Ortuno-Lizaran et al. (2018a) showed the p-α-syn deposits in retinal ganglion cells or intrinsically photosensitive ganglion cells in patients with PD. As a-Syn may spread from neuron to neuron, the a-synuclein may rely on the long axons of the GCs to spread from the retina and brain (Bodis-Wollner et al., 2014b). However, there is not enough evidence to answer some pivotal questions including whether multifocal initiation of a-Syn pathology exists and how some a-Syn species transmit through neural connections or non-neuronal cells.

**TABLE 2:** Table outlining the features of native and phosphorylated a-synuclein in the retina.

	Subject	Retinal layers	Morphometric analysis	Aggregation propensity or toxicity	References
Native a-synuclein	Non-PD, PD patient	GCL, IPL, INL	Soluble a-synuclein, protein aggregates, Lewy body/neurite	±^a^	Guan et al., 2013; Mailankody et al., 2015; Robbins et al., 2021
Phospho-a-synuclein	PD patient	GCL, IPL, NFL	Protein aggregates, Lewy body/neurite	++^b^	Archibald et al., 2009; Schmidt et al., 2011

*Non-PD, healthy control subject; GCL, ganglion cell layer; INL, inner nuclear layer; IPL, inner plexiform layer; NFL, nerve fiber layer.*

*^a^±: none or low aggregation propensity and toxicity.*

*^b^++: increased aggregation propensity and toxicity.*

### Dopaminergic Deficiency

Considering the demonstrated implication of dopaminergic cells in retina functions like those previously mentioned, an impairment in the retinal dopaminergic system is linked to visual symptoms in patients with PD. In 1988, Nguyen-Legros (1988) described the loss of dopaminergic neurons in the retina, and changes in the ERG, VEPs, and contrast sensitivity were observed. Compared the retinal dopamine content in patients who received levodopa therapy(treatment with the DA precursor levodopa 2–15 h before death)and who had not, Harnois and Di Paolo (1990) showed decreased DA in the retinas of subjects with PD. Furthermore, Ortuno-Lizaran et al. (2020) reported the dopaminergic cell degeneration and the loss of synaptic contacts, revealing a failure in DA cells through gap junctions that are involved in visual function. Interestingly, it has recently been proposed that the retina plays an important role in the regulation of the circadian system and motor function. Some articles have reported the motor impairment and circadian disorders in PD animal models (Willis et al., 2014). In turn, using levodopa in the retina (Willis, 2008) and timed light therapy (Videnovic et al., 2017) [a dopamine release stimulation (Li and Tian, 2017)] showed an improvement of DA cells function, enhancing sleep, mood, and anxiety, and also improved motor function. Therefore, the retinal dopaminergic system is affected in PD and may explain the visual deficits, motor impairment, and circadian rhythm alterations described in patients.

With the development of ophthalmic techniques as mentioned above, updated retinal images in patients with PD exhibit reduced number of dopamine cells and dopamine concentration in the retina (Bodis-Wollner et al., 1987; Bodis-Wollner, 1990; Harnois and Di Paolo, 1990; Price et al., 1992). The measuring of the level of dopamine released by dopaminergic neurons is also beneficial for monitoring retinal morphological changes. Clinical data have demonstrated that dopamine and dopamine transporter (DAT) detected by single photon emission computerized tomography (SPECT) or positron emission tomography (PET) promise to be objective and non-invasive markers to identify and determine retinopathy in PD patients (Nguyen-Legros, 1988; Wojtkowski et al., 2004; Biehlmaier et al., 2007). Also, abnormal transmitter production and atrophy in RGCs and RNFL caused lower dopaminergic cells to be identified using the OCT and be a promising marker to monitor the progression of the disease (Inzelberg et al., 2004). Thus, liked as pathological a-Syn, dopaminergic neurons loss may be the key factors triggering retinopathy in PD. Most experimental studies have shown that the formation and aggregation of a-Syn may induce a time dependent loss of DA neurons in the brain (Giordano et al., 2018). However, the specific pathophysiological mechanism of a-Syn in the retina and its relationship with the level of retinal dopamine remains to be further studied.

### Retinal Ganglion Cells Loss and Retinal Nerve Fiber Layer Thinning

As previously mentioned, the ophthalmological examinations visualize on the surface of the retina, such as the RNFL and the retinal capillaries (attenuation, dilatation, aneurismal, and neovascular). Though it is not certain that all retinal thinning in PD is due to a-Syn aggregation or dopaminergic neuronal loss, the two main pathological hallmarks may damage retina structure (RGCs loss or RNFL thinning), interfere with signal transmission, and hence cause visual dysfunction.

Most non-invasive study of the retina in PD patients concentrated on the correlation of thinning RNFL detected by OCT. Inzelberg et al. (2004) first assessed with OCT and reported the RNFL thinning in patients with PD compared with controls. The results showed a decrease in the thickness of inferior quadrant RNFL near its entry to the optic nerve head (Inzelberg et al., 2004). Since then, it has been identified that the RNFL thickness was significantly thinner in four different quadrants, ranging superior, temporal (Altintas et al., 2008; Moschos et al., 2011), inferior (Inzelberg et al., 2004), and nasal (Shrier et al., 2012) in the retina of participants with PD. Likewise, the RNFL thickness in the macular region of PD was significantly lower than in the control groups (Moschos et al., 2011; La Morgia et al., 2013). Notably, the OCT quantification in macular seems to have a higher diagnostic yield than RNFL quadrants quantification (Polo et al., 2016). Sparkly, most studies revealed a significant thinning of the RNFL in the IRL (Hajee et al., 2009; Cubo et al., 2010; Albrecht et al., 2012) (constituting GCL, IPL, and INL) and in the central 5-mm quadrant of the macula (Albrecht et al., 2012; Garcia-Martin et al., 2014b), while no significant changes in the ORL of the retina (Hajee et al., 2009; Cubo et al., 2010; Adam et al., 2013).

The RNFL, as mentioned above, is formed from the axons of the ganglion cells and constitutes the output neurons of the retina. The thinness of the RNFL, specially GCL thinning, largely leads to decreased ERG responses (Cuenca et al., 2005). When comparing visual hallucination and OCT, Adam et al. (2013) reported that visual hallucinations positively correlate with retinal thinning in patients with PD. On the contrary, an inverse correlation of IPL thickness with central contrast sensitivity was recently observed by Lee et al. (2014), and the correlation in PD is weaker that in control groups.

Moreover, there are studies in the literature reporting a correlation between the extent of the RNFL thinning and duration or severity of PD. For instance, Jiménez et al. (2014) demonstrated RNFL thickness correlation with disease severity, and reported a strong inverse correlation between RNFL thickness and the PD severity measured according to the Unified Parkinson’s Disease Rating Scale (UPDRS) score. The result suggested the decreased RNFL thickness evaluated by OCT may be defined a simple biomarker for the clinical duration and average of PD. Similarly, Garcia-Martin et al. (2014a) demonstrated the more serious impairment of inner retinal layers in the patients with long disease duration, rather than healthy controls and PD with short disease duration.

### Retinal Microvascular and Choroidal Structural Changes

Evidence has implicated the correlation of cerebral small vessel diseases with retinal microvascular changes detected by OCTA. In the Atherosclerosis Risk in Communities (ARIC) Study, Wong et al. (2001) detected that retinal microvascular abnormalities are associated with an increased incidence of stroke. Hughes et al. (2016) investigated associations between cerebral infarcts and white matter lesions and abnormalities of the retinal circulation, such as narrower arteriolar diameter, fewer arteriolar branching, and more tortuous venules. Further, clinical data demonstrated that these microvascular changes have been noted to have increased incidence of PD and retinal structural and functional alteration (Guan et al., 2013; van der Holst et al., 2015). Non-invasive tests of retinal vascular impairment are likely to serve as biomarkers for cerebral vascular changes in individuals with PD.

There are studies in the literature evaluating the retinal microvessel status in individuals with PD. In a prospective study (Kwapong et al., 2018), scholars evaluated macula microvasculature and intraretinal layer thickness using SD-OCT. The result demonstrated decreased microvascular density in retina and reported a strong correlation between RNFL thinning and the retinal microvascular abnormality. Also, Shi and collaborators (Shi et al., 2020) characterized lower retinal capillary density, decreased capillary perfusion density, and fractal dimension using OCTA, suggesting the role of retinal structural changes serving as a surrogate biomarker of cerebral changes in PD. On post-mortem analysis of brain tissue from patients with PD, Guan et al. (2013) also observed reduction in capillary branching, fragmentation of capillary, shortening vascular length, and larger diameter in the substantia nigra, middle frontal cortex, and brain stem nuclei. Recently, a cross-sectional study (Robbins et al., 2021) also compared relevant retinal parameters of individuals with PD and age- and sex-matched controls, found increased choroidal area, increased choroidal luminal area, and decreased capillary plexus vessel density and perfusion density in PD. Therefore, non-invasive retinal imaging, OCT, may detect structural changes in retinal microvascular as a novel technique for assessment and detection of PD.

However, the mechanism of retinal microvascular changes in PD is obscure. One may speculate that blood vessel regression effects retinal circulation network, disturbs energy metabolism and biochemistry functions. Retinal microvascular VD and PFD in individuals with PD, to some extent, may reflect the underlying blood vessel changes in neurodegenerative process of PD (Robbins et al., 2021). Further, comparing vascular regression and pathological features of PD in a-Syn overexpression mouse model, Elabi et al. (2021) observed dynamic changes in retinal microvascular morphology accompanied by a pathological accumulation of α-syn deposit. The result suggests the role of retinal microvascular pathology as an important pathophysiological marker in PD (Elabi et al., 2021). Moreover, early discoveries that the eye consists of unique surface molecules and cytokines, and presents some immune responses similar to those in CNS (Streilein, 2003), so retina may display similarities of microvascular changes to the brain.

## Visual Dysfunctions Associated With Morphological Changes in Retina

The retina, as mentioned above, consists of different neurons, dendrites, and axons, and it is responsible for integrating response to the visual system to the cortex. Clinically, patients with PD often suffer from various functional disabilities in central, peripheral, or visuoperceptual vision. Although the visual system does not exist in isolation, we focus on the retina in this article and discuss the visual disorders associated with retinal dysfunction (Table 3).

**TABLE 3:** Retinal abnormalities in PD patients.

Visual abnormality	Morphological changes in Retina	Retinal mechanism defects	References
Visual acuity	RNFL thinning		Postuma et al., 2015; Gbd 2015 Neurological Disorders Collaborator Group, 2017; Ggbd 2016 Parkinson’s Disease Collaborators, 2018; Santos Garcia et al., 2019; Xu et al., 2019
	Loss RGCs		Postuma et al., 2015; Gbd 2015 Neurological Disorders Collaborator Group, 2017; Ortuno-Lizaran et al., 2018b
	Decreased microvascular density		Ortuno-Lizaran et al., 2018a
Contrast sensitivity	RNFL thinning	①Retinal function in ERGs and VEPs; ②Retinal dopaminergic system impairment and dopamine reduction ③Loss of synaptic contacts between retinal neurons	Ggbd 2016 Parkinson’s Disease Collaborators, 2018
	RGCs loss		Ortuno-Lizaran et al., 2018b; Kim et al., 2019
	Thinning of foveal neural tissues		Friedman, 2004; Reddy et al., 2013
Visual hallucinations	RNFL thinning		Armstrong, 2017; Shi et al., 2020
Color vision	Loss RGCs		Ortuno-Lizaran et al., 2018b

### Visual Acuity

Visual acuity (VA) is an ability to discriminate the details of a stimulus. It has been demonstrated that patients with PD present impaired VA in the prodromal phase of the disease (Jones et al., 1992). Compared with age- and sex-matched healthy people using the standard Snellen chart and computerized test, VA is impaired in individuals with PD. Similarly, Han et al. (2020) confirmed that VA in PD patients was worse than this in control groups. Especially, they also found that the worse VA groups have higher incidence of PD than individuals without visual disability, reflecting the visual disorder is one of the premotor symptoms for PD progression (Han et al., 2020).

Evidence has demonstrated a significant positive correlation between lower visual acuity and thinning RNFL thickness (Satue et al., 2017; Visser et al., 2020; Abd Hamid et al., 2021; Table 3). Sparkly, the thinness in the ganglion cell-inner plexiform layer in PD was strongly correlated with low contrast visual acuity via a comprehensive battery of visual function tests (Murueta-Goyena et al., 2019; Marrocco et al., 2020). Recently, Shi and his colleagues characterized retinal capillary complexity of retina in patients with PD, and found that lower retinal capillary and perfusion densities and capillary complexity was negatively correlated with VA (Shi et al., 2020).

According to the above, dopaminergic neuron cells in retina can release dopamine and contribute to functional VA. Aggregates of misfolded a-synuclein and related retinal dopamine depletion lead to injury to light-adapted vision and VA. Nguyen-Legros noted that altered ERGs and VEPs are identical in PD patients and animal models with damaged dopaminergic retinal system (Wong et al., 1985; Olivier et al., 1986; Bodis-Wollner and Tzelepi, 1998; Afsari et al., 2014; Bonilha et al., 2015; Mammadova et al., 2019). In Archibald et al.’s (2011) research, 64 with PD, 26 with PD dementia (PDD), and 32 normal were evaluated using a series of diagnostic procedures about function on vision, cognition, and related pathology. The study reported the impairments in acuity in patients with PD or PDD, and poorer visual acuity in the last stage of untreated patients (Archibald et al., 2011). Likewise, Richard and his members beat out the correlation between poor VA and the lack of dopamine in the retina, but also the better acuity in PD patients receiving drugs (Jones et al., 1992).

### Contrast Sensitivity

Contrast sensitivity is a special vision function involving in the regulation of visual resolution ratio and vision at a variety of spatial and light-black frequencies. Patients with diminished vision contrast sensitivity commonly are susceptible to falls, reading problems, and dark-adapted difficulty. In the 1980s, impaired contrast sensitivity was documented in PD patients in comparison with age-matched controls (Regan and Neima, 1984). Since then, an increasing number of studies have consistently offered proof for the impairment of contrast sensitivity in patients with PD (Langheinrich et al., 2000; Silva et al., 2005; Archibald et al., 2009; Armstrong, 2017). Current studies reported by Polo et al. (2016), showed that contrast sensitivity deficit is more common and severe than other visual disorders. Additionally, the impaired contrast sensitivity was associated with VH and cognitive impairment, as a useful biomarker in patients with PD (Diederich et al., 1998; Ridder et al., 2017).

Like VA, evidence revealed that the inner retinal thinning enhances the presence of contrast sensitivity (Pinkhardt et al., 2020), and the progressive changes in RNFL were associated with progression of abnormal visual function (Satue et al., 2017). Moreover, several studies demonstrated the correlation between impaired vision and remodeled foveal pit, showing the correlation of contrast sensitivity deficit with retinal parafoveal thickness (Miri et al., 2016; Pinkhardt et al., 2020).

In pathological mechanisms, it has been indicated that dopaminergic system impairment in the retina may explain and be partially responsible for the reduced contrast sensitivity in patients with PD. Under physiological conditions, the contrast sensitivity and color vision are mainly modulated through D1 and D2 receptors differentially located in the retinal structure. When these receptors lack activation, there are the dispersion of visual signals and alterations in color vision and contrast sensitivity (Hajee et al., 2009). Also, dopamine reduction may result in loss of synaptic contacts with photoreceptor cells and mRGCs and to disturb contrast sensitivity (Hindle et al., 2013; Kaur et al., 2015; Ortuno-Lizaran et al., 2020). Bulens et al. (1987) reported, contrast sensitivity function of 10 patients with PD before and after levodopa treatment. The remission of contrast sensitivity deficit after the exogenous supplement of dopamine demonstrated the function of the retinal transmitter on the visual pathways. An updated study showed that dopaminergic system impairment and dopamine reduction may be responsible for the reduced contrast sensitivity in PD (Ortuño-Lizarán et al., 2020). In addition, these alterations are linked to loss of dopaminergic synaptic contacts or decline in mRGCs that contribute to circadian rhythm and sleep.

### Visual Hallucinations

Visual hallucination (VH) is a specific feature of PD compared with other Parkinsonian disorders, accounting for 30–40% patients with the disease (Onofrj et al., 2007). Clinically, VH manifests various complex symptoms, including flashes of light, visual perception deficit, and color and motion perception impairment. Most studies in patients with VH demonstrated the disorder was associated with cortical visual discrimination involving in the changes at different visual pathways as well as other neural systems and motor function (Uc et al., 2005). It can explain why VH is also common in cognitive flaw PD patients, and the patients with dementia have the higher prevalence of VH than patients without dementia (Archibald et al., 2011). However, the underlying pathophysiological mechanisms are still unclear. Based on a hypothesis of VH titled the Charles Bonnet syndrome (Stebbins et al., 2004), we know that retinal damage is linked to poor signals in the brain regions, and lead to less visual cortical activation and play a crucial role in the neuropathophysiological function of VH in PD. Moreover, emerging studies stated that defective visual information processing involvement has been demonstrated in VH of PD patients, so retinal impairment appears to be one of mechanisms of the sign (Visser et al., 2020).

To date a limited number of studies reveals that VH appears to be associated with inner retinal thinning. Lee et al. (2014) identified RNFL thinning among the PD subgroups, and noted that RNFL thickness is thinnest in groups without dementia, suggesting RNFL thinning was associated with the occurrence of VH in PD. Similarly, a recent report confirmed the relationship between RNFL thinning and the presence of VH, revealing that individuals with VH had a thinner GCL-IPL than individuals without VH (Visser et al., 2020). It is believed that the old age, disease’s duration, motor disorders, and other non-motor disturbances could worsen the VH as the risk factors in PD (Fénelon et al., 2000; Zhu et al., 2013).

Consistent with visual acuity and contrast sensitivity, retinal pathological changes contribute to the occurrence and development of VH. The dopaminergic system damages have been considered as the pathological basis of VH. Dopamine replacement therapy also supported the evidence for the effects of dopaminergic deficiency on the VH (Onofrj et al., 2002; O’Donnell et al., 2006). Like as the loss of DA, the LB pathology is also associated with the occurrence and progress of VH. Moreover, a number of studies have been proposed to explain VH in PD, noting that reduced levels of γ-aminobutyric acid (GABA) are associated with mechanisms of VH (Firbank et al., 2018).

### Color Vision

As is known to all, color vision is the basic function mediated by the photoreceptor cones in the retina and related visual cortex via some specific visual pathways. In the early stages of PD, patients’ color vision is impaired and deteriorates with disease duration (Price et al., 1992; Buttner et al., 1995). In the 1990s, scholars assessed the color vision in 35 patients with PD and 26 controls, and reported significant abnormality of color vision in PD compared to the healthy people (Price et al., 1992). Recent studies showed the color detection dysfunction affected movement. For instance, Penedo et al. (2018) reported the ability of obstacle avoidance was impaired due to the impairment of color. Likewise, the axial motor impairments were associated with the changes in color discrimination (Bohnen et al., 2017), suggesting shared pathophysiology between the alteration of color vision and motor or mobility dysfunctions in PD. Additionally, the disorders of visual pathways in PD patients might contribute to the occurrence of poor color vision, such as depressive symptoms (Li et al., 2018), idiopathic rapid eye movement sleep behavior disorder (Postuma et al., 2009), and other different retinal areas.

Likewise, RGCs loss and RNFL thinning may be also causes of impaired color vision in PD (Polo et al., 2016). In an observational cross-sectional study, Polo et al. (2016) evaluated visual dysfunction and its correlation with morphological changes in the retina in participants with PD, and found color vision was associated with most GCL thinning while not significantly correlated between RNFL thickness and other visual dysfunction.

As a pathological hallmark, dopaminergic deficiency in the retina showed an association with the impairment in color vision. In mammalian retinal layers, some dopaminergic receptors are in charge of color vision and contrast sensitivity, so alterations in these functions could be the result of lack of dopaminergic systems (Hajee et al., 2009). In patients with PD, deficiency in color vision influences retinal evoked potentials, reflecting abnormalities in dopaminergic synaptic activity in the retina (Bodis-Wollner et al., 1982). Silva et al. (2005) found color visual deficits within the parvo, Konio, and magnocellular pathways in the retina, especially the parvocellular pathway. Meanwhile, they also found the reduction of dopaminergic neurons around the fovea, suggesting that the dysfunctions of these pathways are possibly related to altered dopaminergic modulation.

## Future Perspectives

The increasing number of research explores morphological changes associated with retinal dysfunction in PD as summarized in Table 4. This evidence provides insight into the mechanism underlying visual dysfunctions and retinal changes in PD, mirroring PD brain pathology. Thus, morphological changes or dysfunction in the retina are regarded as a potential approach to diagnosis and monitor Parkinson’s disease, and the successful use of the retinal technology in clinical trials is valid and reliable tools to explore neuropathies in the CNS. However, molecular changes and neuropathological mechanisms involved in retinal changes are obscure. More studies are needed to further validate the significance of retinal pathology and vision deficit to establish the causality of these relationships in PD.

**TABLE 4:** Retinal abnormalities in PD animal models.

Animal	Model	Morphological changes	Retinal defects	References
Rat	Rotenone-induced	Decreased number of RGCs and DACs; INL and ONL thinning	Decreased scotopic and photopic a- and b-waves; Increased b-wave implicit time	Tamer et al., 2005; Onofrj et al., 2006
	6-OHDA-induced	Decreased DA levels	–	Onofrj et al., 2006
Mouse	MPTP-induced	Decreased number of DACs	Reduced oscillatory potentials, a- and b-waves;	Williams et al., 2008
	Retinal a-Syn overexpression	Decreased number of DACs	Decrease of light-adapted ERG responses and visual acuity	Guo et al., 2018
	Prnp- A53T- SNCA	Accumulation of α-synuclein, loss of photoreceptor cells		Weil et al., 2016
	TgM83 (A53T α-synuclein mutation)	Accumulation of a-Syn and phosphorylated tau, decreased number of photoreceptors	–	Weil et al., 2016
	DJ-1 knockout	RPE thinning, decreased number of dopamine	Increased amplitude of b-wave and ERG,	Uc et al., 2005
Rabbit	MPTP-induced	Decreased dopamine level	Decreased amplitude of b-waves and oscillatory potentials	Bertrand et al., 2012
	6-OHDA-inducd	Decreased dopamine level	Decreased amplitude of b-waves	Ortuno-Lizaran et al., 2020
Monkey	MPTP-induced	Decreased number of DACs, Deteriorated postsynaptic neurons	–	Esteve-Rudd et al., 2011
	MPTP-induced	Decreased number of DACs, RNFL thinning	Abnormal VEP and PERG responses	Willis et al., 2014
	6-OHDA-inducd	Decreased number of DACs	Abnormal PERG responses	
Drosophila	a-Syn over-expression	–	Decreased PERG responses	Willis, 2005
	LRRK2-G2019S	Loss of photoreceptor function	Decreased ERG response, loss of visual function	Toscano-Tejeida et al., 2016; Pineda-Rodriguez et al., 2017

## Author Contributions

TT and YY conceived the conception and design of the manuscript. YZ drafted the manuscript. TT was responsible for the revision of the article. YZ and XZ participated in the discussion about the article. All authors read and approved the final manuscript.

## Conflict of Interest

The authors declare that the research was conducted in the absence of any commercial or financial relationships that could be construed as a potential conflict of interest.

## Publisher’s Note

All claims expressed in this article are solely those of the authors and do not necessarily represent those of their affiliated organizations, or those of the publisher, the editors and the reviewers. Any product that may be evaluated in this article, or claim that may be made by its manufacturer, is not guaranteed or endorsed by the publisher.
